# General practitioners' views and experiences of counselling for physical activity through the New Zealand Green Prescription program

**DOI:** 10.1186/1471-2296-12-119

**Published:** 2011-11-02

**Authors:** Asmita Patel, Grant M Schofield, Gregory S Kolt, Justin WL Keogh

**Affiliations:** 1 Centre for Physical Activity and Nutrition Research, Auckland University of Technology, Auckland, New Zealand; 2 School of Science and Health, University of Western Sydney, Sydney, Australia; 3 Faculty of Health Sciences and Medicine, Bond University, Gold Coast, Australia

## Abstract

**Background:**

Regular physical activity is beneficial in both the prevention and management of chronic health conditions. A large proportion of adult New Zealanders, however, are insufficiently active. To help increase population levels of physical activity in New Zealand the Green Prescription, a primary care physical activity scripting program, was developed. The primary aim of this study was to identify why general practitioners (GPs) counsel for physical activity and administer Green Prescriptions. A secondary aim was to examine GPs' views and experiences of Green Prescription counselling for the management of depression.

**Methods:**

Individual face-to-face interviews were conducted with 15 GPs. All interviews were audio-taped and transcribed. Data were analysed using an inductive thematic approach.

**Results:**

Several themes and sub-themes emerged from the data. Notably, GPs counselled for physical activity and prescribed Green Prescriptions for both primary preventive (e.g., weight control) and secondary management (e.g., diabetes management) purposes. GPs reported the benefits of the Green Prescription centred around two main themes: (i) a non-medication approach to a healthier lifestyle and (ii) the support benefits of physical activity. Time constraints within the consultation was the only main theme that emerged regarding the barriers GPs perceived to Green Prescription use. Physical activity in general, and physical activity prescribed through the Green Prescription, were also viewed by GPs as beneficial for the management of depression.

**Conclusions:**

The results of this study suggest that New Zealand GPs view the Green Prescription program as beneficial for their patients with pre-existing conditions and/or weight problems. While this is encouraging, the Green Prescription may also be used to promote physical activity in currently healthy but low-active and sedentary individuals. Such individuals are currently disease free, but are at risk for future health-related problems because of their inactive lifestyle. It is recommended that time constraints of the consultation in regard to administering Green Prescriptions could be dealt with by delegating the more time consuming tasks to the patient support counsellors that support the Green Prescription program, and having practice nurses assist in the administration of Green Prescriptions. Green Prescription counselling in conjunction with antidepressant medication may be beneficial for the management of depression and warrants further research.

## Background

Engagement in regular physical activity can provide both physical and psychological health benefits [1,2]. Increasing focus has now been placed on the psychological benefits of physical activity engagement, especially in the treatment and management of depression. By the year 2020, depression is projected to be the second leading cause of illness and disability [3]. There is some evidence that physical inactivity is a risk factor for depression in adults, including older adults [4-7]. A number of studies that have employed a physical activity intervention with clinically depressed adults have found an association between physical activity engagement and a reduction in depressive symptomatology, or an increase in positive mood [8-11].

A high proportion of the adult New Zealand population remains inactive, achieving less than the recommended level of physical activity per week that is required for health gain [12]. A recent population-based survey indicated that less than one half of adult New Zealanders (48%) meet national physical activity guidelines, with 31% of the adult New Zealand population engaging in some physical activity, and 13% found to be inactive [12].

One approach to assist in increasing population levels of physical activity in New Zealand is the Green Prescription program. The Green Prescription is a public health intervention that was developed and launched in the late 1990's. It is based on national guidelines of achieving 30 minutes of moderate intensity physical activity on five or more days of the week [13]. The Green Prescription is administered in the same way as pharmaceutical treatment, via a prescription. A General Practitioner (GP) or practice nurse prescribes physical activity (e.g., a 30 minute session of daily walking, swimming, tai chi etc) primarily for low-active and sedentary individuals who have a stable medical condition, and secondarily for disease-free individuals who are low-active or sedentary [13,14].

A Green Prescription lasts for a 3-month period, during which time the individual receives a monthly phone call from a patient support counsellor (a trained physical activity specialist). Telephone counselling is based on the transtheoretical model of behaviour change [15], and is aimed to provide the individual with ongoing external support for physical activity. The patient support counsellor helps the individual set realistic goals for physical activity and helps identify solutions for participants regarding their primary barriers to physical activity [13]. A number of randomised controlled trials have demonstrated that the Green Prescription is an efficacious intervention in relation to increasing physical activity and improving various aspects of health in previously low-active and sedentary adults [16-19].

The primary care practice setting provides an ideal environment for physical activity promotion as adult New Zealanders visit their GP on a regular basis. It is estimated that 80% of New Zealand adults and 90% of those aged 65 years and older visit their GP at least once a year [20,21]. Further, GPs are viewed by their patients as being credible sources of health promotion advice [22-24].

Despite the potential for physical activity counselling in general practice, research shows that New Zealand GPs prescribe physical activity at half the prevalence of their counterparts in countries such as Australia and the United States [20]. Population survey data show that 13% of New Zealand adults reported receiving physical activity advice from their GP in the previous 12 months, with 3% reported receiving a Green Prescription within the same time frame [20].

If the Green Prescription is to improve the health and well-being of more New Zealanders, a greater understanding of GPs' perceptions regarding the benefits and barriers to this program would be useful. Limited research exists that has focused on New Zealand GPs' views and experiences of physical activity counselling in general, and more specifically their views and utilization of the Green Prescription program. Therefore, the primary aim of the present study was to identify the reasons GPs counsel for physical activity and administer Green Prescriptions. A secondary aim was to examine GPs' views and experiences of Green Prescription counselling for the management of depression.

## Methods

This study was part of the larger Healthy Steps study, an investigation of the effectiveness of Green Prescriptions in increasing physical activity and quality of life in low-active, community-dwelling older adults [25-27].

### Participants

Participants were 15 GPs (10 female and 5 male) from the Auckland region of New Zealand. Participants ranged in age from 36 to 64 years of age (mean age = 50.8 years, SD = 7.1 years). Participants had been practising medicine in general practice settings between 1 and 30 years (mean = 22.1 years, SD = 10.3 years). Each participant was engaging in a minimum of 150 minutes of moderate-intensity physical activity per week. The majority of participants were engaging in daily recreational walking, with extra activities being undertaken on most weekends (e.g., kayaking, tramping, swimming). There was a range in the frequency in which participants issued Green Prescriptions. Nine participants were categorised as regular users (i.e., issuing at least one Green Prescription per week), two participants were categorised as sometime users (i.e., issuing at least one Green Prescription per month), two participants were categorised as infrequent users (i.e., issuing a Green Prescription once every few months), and two participants had stopped issuing Green Prescriptions.

### Measure

A structured interview schedule comprising open-ended questions was developed for this study based on relevant literature relating to physical activity prescription and Green Prescription use. The interview schedule (see Table 1) acted as a guide for the interviewer and ensured that all participants were asked the same questions. The focus of the interview was on GPs' views and experiences of the Green Prescription program, specifically, what GPs perceived to be the benefits and barriers of Green Prescription use. The questions contained in the interview schedule were open-ended and designed to allow for discussion and elaboration. This allowed the interviewer to follow-up on a participant's response to a particular question (e.g., to gain clarification or to ensure more discussion about a particular issue or topic that a participant had raised). Questions contained in the interview schedule were divided into three specific topic areas: (1) those relating to physical activity advice in the primary practice setting, (2) those relating to Green Prescription counselling and, (3) those relating to Green Prescription use for the management of depression. Although the interview schedule was based on set questions, ample opportunity was provided for participants to raise other related issues during the interview.

**Table 1:** Interview questions

Topic Area	Questions
**Physical activity advice in the primary care setting**	Why do you give out physical activity advice to your patients?
**Green Prescription counselling**	What do you feel are the benefits of Green Prescription use?
	As a GP what barriers do you encounter when you are thinking about writing out a Green Prescription for a patient?
	There are differing thoughts in relation to whether a Green Prescription is designed more to prevent a condition or to manage a condition, what are your thoughts?
**Green Prescription counselling for depression**	What are your thoughts on Green Prescriptions being written for mental health conditions such as depression?

### Procedure

Participants were recruited through the University of Auckland's General Practitioner Database. Recruitment of participants was based on geographical location. An equal number of potential participants from North, East, West, and South Auckland were sent a letter of invitation and an information sheet detailing the study and providing the researchers' contact details. A total of 80 letters of invitation were mailed out to potential participants to obtain 15 positive responders. Those who were interested in participating replied by fax or telephone and an interview time was arranged. Participants were interviewed in their place of work (the general practice setting). Interviews took between 20 and 30 minutes to complete. All interviews were audio-taped for later transcription and data analysis. Informed written consent was obtained from each participant. The study was approved by Auckland University of Technology's Ethics Committee.

### Data Analysis

A qualitative methodology was employed for this study. All interviews were audio-taped and transcribed verbatim. Data were analysed using an inductive thematic approach based on Auerbach and Silverstein's [28] approach to thematic analysis. The first step in the data analysis involved reading and re-reading the transcripts (e.g., raw text) several times for each question within one of the three topic areas: (1) physical activity advice in the primary care practice setting, (2) Green Prescription counselling and, (3) Green Prescription use for the management of depression. The second step involved identifying repeating ideas within a particular topic area. This process involved focusing on text where several of the GPs used similar words or experiences to convey the same idea. From a focus on repeating ideas, coding and specific themes (e.g., an organisation of repeating ideas that is given a specific name that communicates what participants are trying to convey) emerged from the raw data. Coding and themes were verified by all members of the research team (peer triangulation). This process helped reduce individual researcher bias.

## Results

Data were examined under the three broad headings mentioned above. For each topic area, main themes and associated sub-themes are outlined and direct quotes are included that illustrate specific points, or that highlight expressed views or experiences encountered. Table 2 lists the themes and sub-themes as well as provides representative quotes for each of the topic areas.

**Table 2:** Topic areas, themes and sub-themes

Topic Area	Themes	Sub-themes	Sample Quotes
General physical activity advice within the primary care setting	Pre-existing conditions and weight management		"I do try and encourage physical activity for a number of reasons: for stress management, for weight control, for blood pressure control" (GP 11)
Green Prescription counselling	GPs perceived benefits of the Green Prescription program	(i) A non-medication approach to a healthier lifestyle	"The great thing about it is that you are not stuffing some medicine into them" (GP 8)
		(ii) The support benefits of physical activity	"I think that one of the big benefits would be that patients get much better exercise advice, and more prolonged support than they would get from a GP" (GP1)
	GPs perceived barriers to Green Prescription use	(i) Time-constraints of the consultation	"Time! Because patients generally have quite complex problems, and multiple problems" (GP 7)
	Administering Green Prescriptions	(I) Preventive purposes	"People who have a family history of diabetes, or who are hypertensive, or are starting to get obese. Pushing hard for them to exercise, you're going to prevent things from happening" (GP2)
		(ii) Management purposes	"To treat conditions like diabetes, or to help people manage them" (GP 2)
Green Prescription counselling for the management of depression	Perceived physical benefits of increased physical activity		"I think there's nothing like getting out in fresh air and going for a little bit of a walk. Because you know it increases endorphins. You feel better" (GP 2)

### General physical activity advice within the primary care setting

#### Theme: Pre-existing conditions and weight management

Pre-existing conditions and weight management was the only theme that emerged regarding why general verbal advice for physical activity is given by GPs in daily consultations. This theme illustrated how GPs view physical activity as a form of secondary management for patients who have pre-existing conditions (e.g., type 2 diabetes, hypertension, heart conditions). This theme also highlighted that GPs view physical activity as beneficial in the maintenance of healthy body weight. This was demonstrated by comments such as:

*"For somebody who's hypertensive or overweight, diabetic. High risk team of people." *(GP 11)

*"I do try and encourage physical activity for a number of reasons: for stress management, for weight control, for blood pressure control." *(GP 9)

"Sometimes if they come in for a pill repeat and if I think they are highly overweight, then I go through it with them as part of the consultation." (GP 11)

*"A concern regarding their weight, and if they have co-morbidities, we definitely touch on that briefly." *(GP 14)

### Green Prescription Counselling

Three main themes and five associated sub-themes emerged within this topic area. These were associated with GPs' perceived benefits and barriers to Green Prescription use, and the reasons for administering Green Prescriptions.

#### Theme: GPs' perceived benefits of the Green Prescription program

Two main associated sub-themes emerged: (i) a non-medication approach to a healthier lifestyle, and (ii) the support benefits of physical activity.

#### Sub-theme: A non-medication approach to a healthier lifestyle

A majority of GPs emphasised that one of the most salient benefits of Green Prescription use is that it is a drug-free process. Some GPs discussed how a Green Prescription gives importance to physical activity as a valid treatment for health gain, as it is endorsed by GPs and it is presented in the same format as prescription medication. The following quotes highlight these points:

*"The great thing about it is that you are not stuffing some medicine into them." *(GP 8)

*"It shows them that you think exercise is important. So reinforcing that not just pills are going to make people better." *(GP 4)

*"It officializes the fact that you think that exercise is a treatment by putting it on a bit of paper. Gives your stamp of approval to it. It means it's important to us." *(GP 1)

#### Sub-theme: The support benefits of physical activity

Both the prolonged and specialised support and counselling that patients received from the Green Prescription patient support counsellor was seen as beneficial by most GPs. GPs viewed the patient support counsellor as having both the time and skills to fully support patients in initiating and maintaining their physical activity or exercise. Some GPs discussed how time constraints of the consultation can hinder such counselling in the practice setting. GPs also stressed how the specialised support provided by the counsellor was important in that it increased patient safety and allowed monitoring of activity levels. The following quotes illustrate these points:

"I think one of the big benefits would be that patients get much better exercise advice and more prolonged support than they can get from a GP." (GP 1)

"I see the Green Prescription as really important. To have that support - that is really important for helping people make the behaviour change that is necessary for it to become an ongoing lifestyle, rather than just a short term change." (GP 13)

"People do it in a grade and methodological fashion. It's safe." (GP 3)

#### Theme: GPs perceived barriers to Green Prescription use

Time constraints of the consultation was the only main theme that emerged in relation to GPs' perceived barriers to Green Prescription use.

#### Sub-theme: Time constraints of the consultation

The majority of GPs stated that time constraints of the consultation was the most salient barrier for them in relation to administering Green Prescriptions. GPs discussed how some patients presented with multiple problems or conditions, and how this left little or no time for physical activity counselling, or specifically administering a Green Prescription. The following quotes illustrate this:

*"Time! Because patients generally have quite complex problems and multiple problems." *(GP 7)

*"There was a time barrier to discuss physical activity with them. Ten or fifteen minutes for the consultation. Not a lot of time." *(GP 11)

*"It is important, so you generally squeeze it in. I tended to run over time." *(GP 13)

A number of the GPs attempted to deal with the time barrier in a number of ways. A couple of GPs mentioned that in some cases their practice nurse also administered Green Prescriptions. Some GPs delegated the more time consuming tasks (such as choosing an activity) to the patient support counsellor. The following quotes demonstrate this:

"I actually will hand some of these patients onto the nurse, and say, look could you also look at diet, exercise, Green Prescription." (GP 13)

"Sometimes we had nurses who were keen and supportive." (GP 13)

"Time is the main barrier, just one more thing to do. I delegate a lot of the time consuming part to the Green Prescription people." (GP 1)

#### Theme: Administering Green Prescriptions

Two main sub-themes emerged: (i) preventive purposes, and (ii) management purposes.

#### Sub-theme: Preventive purposes

A Green Prescription was issued by the GPs for primary preventive purposes when there was an awareness of a family history for a certain condition. Also, if a patient was overweight, a Green Prescription was viewed by some GPs as a preventive measure, to lessen the chance of developing chronic diseases (e.g., diabetes). Patients who had high blood pressure were also seen as ideal candidates for a Green Prescription intervention. The following quotes highlight this:

*"I'm dealing with people who might get problems. People who have a family history of diabetes, or who are hypertensive, or are starting to get obese. Pushing hard for them to exercise, you're going to prevent things from happening." *(GP 2)

*"For diabetes prevention for those at risk. Patients at risk for ischaemic heart disease, or maybe hypertension, or high cholesterol. It's worthwhile for a Green Prescription and exercise programmes." *(GP 15)

#### Sub-theme: Management purposes

GPs also addressed how they administer Green Prescriptions to help manage certain conditions. A Green Prescription was seen as helpful in managing pain for patients with arthritis. GPs also discussed how they have issued Green Prescriptions for weight control management for patients who have diabetes. The following quotes convey that GPs have found physical activity and exercise to be a valid form of management for certain conditions. The following quotes demonstrate this:

*"I'm dealing with people who've already got problems. To treat conditions like diabetes, or to help people manage them." *(GP 2)

*"It can be useful to manage symptoms like pain and arthritis." *(GP 3)

*"If they are overweight, they'll lose weight, that's got to be helping them with their current condition." *(GP 7)

### Green Prescription Counselling for the Management of Depression

#### Theme: Perceived physical benefits of increased physical activity

The perceived physical benefits of increased physical activity was the one main theme that emerged regarding Green Prescription use for the management of depression. All

15 GPs emphasised the importance of physical activity engagement because of the natural biochemical processes that occur from such activity. GPs tended to discuss how patients could help themselves by engaging in physical activity to release endorphins, to enhance positive mood. The following accounts illustrate this:

*"We know that exercise is very good for depression, from the natural endorphins to socialisation." *(GP 12)

*"I think there's nothing like getting out in fresh air and going for a little bit of a walk, because you know it increases endorphins. You feel better." *(GP 2)

*"You say, now if you want to get better faster, what you want to do is increase your own endorphins." *(GP 6)

*"I tell them that, exercise alone will raise your serotonin, and that will make you feel better if you do it every day." *(GP 15)

Some GPs also discussed how they have used Green Prescription counselling in conjunction with antidepressant medication to manage patients with depression. Physical activity engagement in general, as well as through the Green Prescription was viewed by some GPs as helping lessen the need for drug treatment, or lessening the dosage of antidepressant medication. The following quotes demonstrate these points:

*"The more exercise they do the less their depression, and the less medication. I've never regretted giving a patient with depression an exercise prescription. They definitely do better." *(GP 8)

*"It's clearly shown if they exercise they get a much better clinical response, and that relates to serotonin lift when they exercise. So serotonin uptake of the medication of fluoxetine." *(GP 15)

## Discussion

This study examined the reasons GPs provided verbal advice for physical activity and administered Green Prescriptions. Both general verbal advice for physical activity and Green Prescriptions were administered for the same two reasons; either for preventive purposes or for management purposes. Green Prescriptions were administered as a form of primary prevention for patients who were currently disease free, but due to their weight (i.e., overweight or obese) were at risk for developing future health-related problems [20,29,30]. This finding is consistent with those from the annual New Zealand Sport and Recreation surveys which monitors Green Prescription utilization. These surveys have shown that Green Prescriptions are issued for weight management purposes more than for any other condition [31-33].

While GPs in the present study were administering Green Prescriptions or providing verbal advice for physical activity as a form of primary prevention for weight management purposes, they were not administering Green Prescriptions or verbally recommending physical activity for patients who were low-active or sedentary, but currently disease free and of average body weight. Sedentary individuals can benefit from a Green Prescription (or from verbal advice to become physically active), as they are currently disease free, but are at risk for future non-communicable diseases and conditions because of their inactive lifestyle [20,30,34].

It has been argued [20,35] that some GPs believe that physical activity prescription or advice is likely to be more effective when it is linked to an existing chronic health condition. The Green Prescription is used more as a form of management for pre-existing conditions than as a form of prevention. However, low-active and sedentary individuals who are of normal body weight and are disease-free are not likely to see their GP on a regular basis, and thus, are less likely to be counselled for physical activity. This is one such example of a systemic barrier to implementation for GPs who administer Green Prescriptions.

A non medication approach to a healthier lifestyle was identified by GPs as being a salient benefit of the Green Prescription program, as patients did not encounter negative side effects that drug use entails. GPs discussed how in the eyes of patients a Green Prescription validates the importance of physical activity as a treatment for health gain. This was related to physical activity advice being provided by a medical professional. Such a view is consistent with the research demonstrating that some groups of individuals are more likely to consider and adhere to physical activity advice when it is imparted by their GP [22,36].

A Green Prescription also validates the importance of physical activity as a treatment for health gain as it presented in a prescription format like pharmaceutical treatment. Research shows that the Green Prescription format can be understood by many individuals, because the use of a prescription is a well understood interaction between doctor and patient [17,19]. Prescriptions are viewed by individuals as being sources of medical help, with a written prescription for physical activity also acting as a tangible reminder for the patient to engage in activity [17,19,37].

The prolonged and specialised support that patients receive from the Green Prescription patient support counsellor was identified by GPs as being another benefit of the Green Prescription program. This finding is important as previous research has not specifically focused on the aspects of the Green Prescription program that GPs believe best facilitate ongoing physical activity in their patients. In annual Sport and Recreation Green Prescription surveys, patients have stated that the advice, support, and encouragement that they have received from their patient support counsellor has helped to keep them active and complete their Green Prescription [30,38]. The GPs views on the importance of the patient support counsellor is also consistent with the results of a qualitative study investigating patients' views and experiences of receiving a Green Prescription [36]. Some patients in the Elley et al. [36] study viewed their patient support counsellor as a "significant other" - someone who had some influence over their behaviour. For example, some patients felt motivated to comply with their Green Prescription because of the ongoing telephone support they received from their patient support counsellor.

Time constraints of the consultation were cited as the most salient barrier to Green Prescription use by GPs, a finding consistent with that of earlier studies [37,35]. The present study is one of a few studies that have specifically examined how New Zealand GPs have dealt with their perceived barriers of the Green Prescription program. One GP mentioned that she delegated the more time consuming tasks, such as choosing an activity, to the Green Prescription patient support counsellor. In some practices, practice nurses also assisted in the administering of Green Prescriptions. These two methods to reduce time demands are important given that both New Zealand and international literature consistently cites time constraints as GPs' most salient barrier to physical activity counselling [35,37,39-41]. There is some evidence to suggest that in some cases it can be less time consuming for a GP to outline the Green Prescription program and administer a Green Prescription than to start a patient on a new medication [42]. If further studies can replicate the findings of Wyndard [42], it is hoped that even more GPs will see the promotion of the Green Prescription as a time-efficient and beneficial intervention for many of their patients.

This is the first New Zealand based study that has examined GPs views on the role that the Green Prescription program can have in helping manage depression. In the present study, all GPs viewed physical activity engagement in general, as well as prescribed physical activity through the Green Prescription program as beneficial for the management of depression. GPs stressed that this was related to the release of natural mood enhancing chemicals (e.g., endorphins). Some GPs discussed how they use Green Prescription counselling in conjunction with antidepressant medication. This combined treatment mode has been studied in randomised controlled trials [43,44]. One study [43] found physical activity to be just as effective as antidepressant medication in decreasing depressive symptomatology over the course of the 16-week intervention period in individuals who had a clinical diagnosis of depression. However, this study did not have a follow-up period to ascertain the long-term effect that physical activity had on depressive symptomatology.

A growing body of research has demonstrated that some degree of regular physical activity or exercise can lower depressive symptomatology in adults who have a clinical diagnosis of depression [9-11,43-47]. Physical activity interventions may be more beneficial in the management of depression for some groups of individuals (i.e., older adults) compared to antidepressant medication [48]. Physical activity interventions are less likely to have adverse 'side effects' and can be undertaken on a regular, long-term basis. Compared to drug treatment, there is also no negative stigma attached to engaging in regular physical activity [49].

### Strengths and Limitations

A main strength of this study is that a qualitative methodology was employed to allow GPs to voice their views and experiences of counselling for physical activity in general, and more specifically, through the Green Prescription program. This allowed a range of issues to be discussed and themes to be derived from the data. A potential limitation of this study was the relatively small number of GPs interviewed. Thus, generalising findings to the larger GP population may be problematic. Also, participation in this study was voluntary. Potential participants were mailed a letter of invitation that outlined the main aims of the study. Although it is likely that GPs who administered Green Prescriptions and/or gave verbal advice for physical activity would be more interested in participating in this study than GPs who did not, the sample interviewed was diverse in this respect.

## Conclusions

The GPs in the present study acknowledged the health-related benefits of physical activity and were providing physical activity advice and prescription to patients who could most benefit from regular physical activity engagement, such as those who were overweight and those who presented with chronic conditions. Physical activity in general, and physical activity prescribed through the Green Prescription program, was seen as beneficial for the management of depression. More research is required into the role that the Green Prescription can have in contributing to the management of depression. As in the case of previous research, GPs did not mention dispensing Green Prescriptions or verbal advice for physical activity to low-active or sedentary patients if these individuals were of normal body weight and had no chronic conditions. While it is beneficial to use physical activity counselling, including the Green Prescription, as a form of secondary treatment to manage existing conditions, there also needs to be a focus on using physical activity prescription for primary preventive purposes. For example, sedentary individuals who are currently disease free can benefit from a Green Prescription, as they are at risk for future non-communicable diseases and conditions because of their inactive lifestyle. Failure to counsel sedentary individuals is a missed opportunity for primary prevention that will most likely lead to adverse health outcomes for such individuals in the future. Future research needs to focus on the larger structures that can be put into place to help GPs screen their patients for physical (in)activity during the consultation process.

## Competing interests

The authors declare that they have no competing interests.

## Authors' contributions

AP, GMS and GSK contributed to study design. AP collected the data. AP, GMS, GSK and JWLK analyzed the data and drafted the manuscript. All authors read and approved the final manuscript.

## Pre-publication history

The pre-publication history for this paper can be accessed here:

http://www.biomedcentral.com/1471-2296/12/119/prepub

## References

[B1] Bauman A Updating the evidence that physical activity is good for health: an epidemiological review 2000-2003 J Sci Med Sport 2003 7 6 19 10.1016/s1440-2440(04)80273-1 15214597

[B2] Warburton DER Nicol CW Bredin SSD Health benefits of physical activity: the evidence Can Med Assoc J 2006 174 801 809 10.1503/cmaj.051351 PMC1402378 16534088

[B3] World Health Organization Depression. What is depression? 2009 Geneva, Switzerland: World Health Organization

[B4] Brown WJ Ford JH Burton NW Marshall AL Dobson AJ Prospective study of physical activity and depressive symptoms in middle-aged women Am J Prev Med 2005 29 265 272 10.1016/j.amepre.2005.06.009 16242588

[B5] Camacho TC Roberts RE Lazarus NB Kaplan GA Cohen RD Physical activity and depression: evidence from the Alameda County Study Am J Epidemiol 1991 134 220 230 10.1093/oxfordjournals.aje.a116074 1862805

[B6] Lampinen P Heikkinen R Ruoppila I Changes in intensity of physical exercise as predictors of depressive symptoms among older adults: an eight-year follow up Prev Med 2000 30 371 380 10.1006/pmed.2000.0641 10845746

[B7] Strawbridge J Deleger S Roberts RE Kaplan GA Physical activity reduces the risk of subsequent depression for older adults Am J Epidemiol 2002 156 328 334 10.1093/aje/kwf047 12181102

[B8] Bartholomew JB Morrison D Ciccolo JT Effects of acute exercise on mood and well-being in patients with major depressive disorder Med Sci Sports Exerc 2005 37 2032 2037 10.1249/01.mss.0000178101.78322.dd 16331126

[B9] Dimeo F Bauer M Varahram I Proest G Halter G Benefits of aerobic exercise in patients with major depression: a pilot study Br J Sports Med 2001 35 114 117 10.1136/bjsm.35.2.114 PMC1724301 11273973

[B10] Dunn A Trivedi M Kampert J Clark C Chambliss H Exercise treatment for depression, efficacy and dose response Am J Prev Med 2005 28 1 8 10.1016/j.amepre.2004.09.003 15626549

[B11] Harris A Cronkite R Moos R Physical activity, exercise coping, and depression in a 10 year cohort study of depressed patients J Affect Disord 2006 93 79 85 10.1016/j.jad.2006.02.013 16545873

[B12] Sport and Recreation New Zealand Active New Zealand Survey. Sport, Recreation and Physical Activity Participation among New Zealand Adults 2008 Wellington, New Zealand: Sport and Recreation New Zealand

[B13] Sport and Recreation New Zealand Green Prescription Overview 2006 Wellington, New Zealand: Sport and Receation New Zealand

[B14] Pringle R Green Prescriptions: effective health promotion? J Phys Educ N Z 1998 31 7 16

[B15] Prochaska J Marcus B Dishman R The transtheoretical model: Applications to exercise 1994 Champaign Illinois: Human Kinetics Advances in exercise adherence

[B16] Elley CR Kerse N Arroll B Robinson E Effectiveness of counselling patients on physical activity in general practice: cluster randomized controlled trial BMJ 2003 326 793 799 10.1136/bmj.326.7393.793 PMC153098 12689976

[B17] Pfeiffer B Clay S Conaster R A Green Prescription study: does written exercise prescribed by a physician result in increased physical activity among older adults? J Aging Health 2001 13 527 538 10.1177/089826430101300405 11813739

[B18] Lawton BA Rose SB Elley CR Dowell AC Fenton A Moyes SA Exercise on prescription for women 40-74 recurited through primary care: two year randomised controlled trial BMJ 2008 337 a2509 10.1136/bmj.a2509 PMC2769033 19074218

[B19] Swinburn B Walter L Arroll B Tiylard M Russell D The Green Prescription study: a randomised controlled trial of written exercise advice provided by general practitioners Am J Public Health 1998 88 288 291 10.2105/ajph.88.2.288 PMC1508188 9491025

[B20] Croteau K Schofield G McLean G Physical activity advice in the primary care setting: results of a population study in New Zealand Aust N Z J Public Health 2006 30 262 267 10.1111/j.1467-842x.2006.tb00868.x 16800204

[B21] Ministry of Health A Portrait of Health: Key results from the 2006/07 New Zealand Health Survey 2008 Wellington, New Zealand: Ministry of Health

[B22] Booth M Bauman A Owen N Gore C Physical activity preferences, preferred sources of assistance, and perceived barriers to increased activity among inactive Australians Prev Med 1997 26 131 137 10.1006/pmed.1996.9982 9010908

[B23] Lawlor D Keen S Neal R Increasing population levels of physical activity through primary care: general practitioners knowlege, attitudes and self-reported practice Fam Pract 1999 16 250 254 10.1093/fampra/16.3.250 10439978

[B24] Tulloch H Fortier M Hogg W Physical activity counseling in primary care: who has and who should be counseling? Patient Educ Couns 2006 64 6 20 10.1016/j.pec.2005.10.010 16472959

[B25] Kolt GS Schofield GM Kerse N Garrett N Schluter P Ashton T Patel A The Healthy Steps study: a randomized controlled trial of a pedometer-based Green Prescription for older adults. Trial protocol BMC Public Health 2009 9 404 10.1186/1471-2458-9-404 PMC2780414 19878605

[B26] Kolt GS Garrett N Ashton T Patel A Healthy Steps trial: pedometer-based advice improves physical activity for low-active older adults Ann Fam Med in press 10.1370/afm.1345 PMC3354969 22585884

[B27] Leung W Ashton T Kolt GS Schofield GM Garrett N Kerse N Patel A Cost-effectiveness of pedometer-based versus time-based Green Prescriptions: the Healthy Steps Study Aust J Prim Health in press 10.1071/PY11028 23069363

[B28] Auerbach C Silverstein L Qualitative data. An introduction to coding and analysis 2003 New Youk: New York University Press

[B29] Eakin I Brown W Schofield G Mummery K Reeves M General practitioner advice on physical activity-who gets it? Am J Health Promot 2007 21 225 228 10.4278/0890-1171-21.4.225 17375487

[B30] Kreuter MW Scharff DP Brennan LK Lukwago SW Physician recommendations for diet and physical activity: which patients get advised to change? Prev Med 1997 26 825 833 10.1006/pmed.1997.0216 9388794

[B31] Sport and Recreation New Zealand Green Prescription patients survey 2008 Wellington, New Zealand: Sport and Recreation New Zealand

[B32] Sport and Recreation New Zealand Green Prescriptions in general practice 2007 Wellington, New Zealand: Sport and Recreation

[B33] Van Aalst I Daly C Green Prescriptions in general practice 2005 Wellington, New Zealand: Sport and Recreation New Zealand

[B34] Wee C McCarthy E Davis R Phillips R Physician counseling about exercise JAMA 1999 282 2846 2848 10.1001/jama.282.16.1583 10546701

[B35] Swinburn B Walter L Arroll B Tiylard M Russell D Green Prescriptions: attitudes and perceptions of general practitioners towards prescribing exercise Br J General Pract 1997 47 567 569 PMC1313106 9406491

[B36] Elley CR Dean S Kerse N Physical activity promotion in general practice Aust Fam Physician 2007 36 1061 1064 18075637

[B37] Gribben B Goodyear-Smith F Grobbelaar M O'Neil D Walker S The early experiences of general practitioners using Green Prescriptions N Z Med J 2000 113 372 373 11050901

[B38] Van Aalst I Daly C Green Prescription patients 2005 Wellington, New Zealand: Sport and Recreation New Zealand

[B39] Kennedy M Meeuwisse W Exercise counselling by family physicians in Canada Prev Med 2003 37 226 232 10.1016/s0091-7435(03)00118-x 12914828

[B40] McKenna J Naylor T McDowell N Barriers to physical activity by general practitioners and practice nurses Br J Sports Med 1998 32 342 247 10.1136/bjsm.32.3.242 PMC1756103 9773175

[B41] Ribbera A McKenna J Riddoch C Physical activity promotion in general practices of Barcelona: a case study Health Educ Res 2006 21 538 548 10.1093/her/cyl008 16702195

[B42] Wynard R Green Rx still largely overlooked option N Z Doctor 2006 5

[B43] Blumenthal J Babyak M Moore K Craighead E Herman S Khatri P Waugh R Napolitano M Forman L Applebaum M Doraiswamy P Krishan K Effects of exercise training on older patients with major depression Arch Intern Med 1999 159 265 272 10.1001/archinte.159.19.2349 10547175

[B44] Mather AS Rodriguez C Guthrie MF McHarg M McMurdo ME Effects of exercise on depressive symptoms in older adults with poorly responsive depressive disorder Br J Psychiatry 2002 180 411 415 10.1192/bjp.180.5.411 11983637

[B45] Singh NA Clements KM Fiatarone Singh MA The efficacy of exercise as a long term antidepressant in elderly subjects: a randomized controlled trial J Gerontol A Biol Sci Med Sci 2001 56A M497 M504 10.1093/gerona/56.8.m497 11487602

[B46] McNeil K LeBlanc E Joyner M The effect of exercise on depressive symptoms in moderately depressed elderly Psychol Aging 1991 6 487 488 10.1037//0882-7974.6.3.487 1930766

[B47] Penninx B Ztrjrdki ZW Pandya J Miller M Di Bari M Applegage WB Pahor M Exercise and depressive symptoms: a comparison of aerobic and resistance exercise effects on emotional and physical function in older persons with high and low depressive symptomatology J Gerontol B Psychol Sci Soc Sci 2002 57B P124 P132 10.1093/geronb/57.2.p124 11867660

[B48] Phillips WT Kiernan M King AC Physical activity as a nonpharmocological treatment for depression: a review Contemp Health Prac Rev 2003 8 139 152

[B49] Blake H Mo P Malik S Thomas S How effective are physical activity interventions for alleviating depressive symptoms in older people? A systematic review Clin Rehabil 2009 23 873 887 10.1177/0269215509337449 19675114

